# Melatonin Ameliorates Coxsackievirus B3-Induced Myocarditis by Regulating Apoptosis and Autophagy

**DOI:** 10.3389/fphar.2018.01384

**Published:** 2018-12-04

**Authors:** Yimiao Sang, Xiaohong Gu, Lulu Pan, Chunxiang Zhang, Xing Rong, Tingting Wu, Tianhe Xia, Yuechun Li, Lisha Ge, Yuanhai Zhang, Maoping Chu

**Affiliations:** ^1^Children’s Heart Center, The Second Affiliated Hospital and Yuying Children’s Hospital, Institute of Cardiovascular Development and Translational Medicine, Wenzhou Medical University, Wenzhou, China; ^2^Department of Pediatrics, The First Affiliated Hospital of Zhejiang University, Hangzhou, China; ^3^Child Health Manage Department, Maternal and Child Health Care Institution, Wenzhou, China; ^4^Department of Biomedical Engineering, School of Medicine and School of Engineering, The University of Alabama at Birmingham, Birmingham, AL, United States; ^5^Department of Cardiology, The Second Affiliated Hospital and Yuying Children’s Hospital of Wenzhou Medical University, Wenzhou, China; ^6^Department of Pediatrics, The Second Affiliated Hospital and Yuying Children’s Hospital of Wenzhou Medical University, Wenzhou, China

**Keywords:** myocarditis, melatonin, autophagy, apoptosis, coxsackievirus B3

## Abstract

Current therapeutics options for viral myocarditis are unsatisfactory. Melatonin (MLT), a hormone secreted by the pineal gland and other organs, has protective effects on ischemic heart injury. However, the potential therapeutic effect of MLT on viral myocarditis is unknown. In this study, we investigated the protective effect of MLT on viral myocarditis in a mouse model of myocarditis infected with coxsackievirus B3 (CVB3) and explored the probable mechanisms. Mice with CVB3-induced myocarditis displayed inflammatory cell infiltration and interstitial edema. MLT treatment significantly ameliorated the myocardial injuries. In addition, the rate of autophagy changed, although apoptosis was inhibited in mouse hearts following treatment with MLT. These results suggest that MLT has a strong therapeutic effect on acute viral myocarditis, which is associated with changes in autophagy and apoptosis in the heart. Thus, MLT could be a promising novel therapeutic approach against viral myocarditis.

## Introduction

Viral myocarditis can lead to arrhythmia, heart failure, and sudden death (Dennert et al., 2008). Coxsackievirus B3 (CVB3) is believed to be a major trigger of viral myocarditis by inducing myocardial apoptosis and/or necrosis (Hendry et al., 2014). Many therapeutic strategies have been attempted to reverse the underlying active myocardial inflammation. Nevertheless, they have been far from satisfactory in clinical applications (Hendry et al., 2014; Mody et al., 2014; Hughes et al., 2015). Identifying new therapeutic methods based on novel molecular mechanisms of virus-mediated injury of the heart is an emergency need in pediatric cardiology.

Melatonin (MLT; *N*-acetyl-5-methoxytryptamine) is a hormone produced by the pineal gland and other organs. Some studies have found that MLT can act as a protective agent during pathological conditions by influencing a variety of molecular pathways, including inflammation, apoptosis, proliferation, metastasis, and angiogenesis (Maria and Witt-Enderby, 2014; Peschke et al., 2015). Additionally, MLT is a powerful antioxidant (Reiter et al., 2016) and has a protective effect on heart ischemia/reperfusion injury (I/R) and myocardial infarction (Chua et al., 2016; Favero et al., 2016). However, the potential therapeutic effect of MLT on viral myocarditis is currently unknown.

Autophagy plays a key role maintaining cellular homeostasis by recycling damaged organelles and proteins and provides an important function in cellular defense by removing intracellular pathogens, such as viruses, bacteria, and parasites (Jackson, 2015). Studies have shown that autophagy is exploited by RNA viruses, such as CVB3, to facilitate viral replication and to acquire metabolites (Deretic et al., 2013). Thus, autophagy is a critical cellular event associated with viral myocarditis.

Apoptosis is a unique type of programmed cell death (Kerr et al., 1972). The induction of apoptosis occurs through mitochondrial (intrinsic) or death receptor (exogenous) pathways, both of which lead to a common mechanism of cell death (Saraste et al., 2003). Apoptosis is another well-known cellular event that occurs in hearts infected with viral or non-viral forms of myocarditis (Carthy et al., 1998; Yuan et al., 2003).

In this study, we investigated the effects of MLT on myocarditis and assessed whether inhibiting autophagy and apoptosis is involved in MLT-mediated effects on myocarditis in CVB3-infected mice.

## Materials and Methods

### Animals

All animal experiments were approved by the Animal Ethics Committee of Wenzhou Medical University and were in accordance with the NIH Guidelines for the Care and Use of Laboratory Animals. A total of 150 BALB/c male mice (5 weeks old, weight 20.5 ± 2 g) were purchased from the Experimental Animal Research Center (Zhejiang, China) and were randomly assigned to five groups: (i) normal (*n* = 20); (ii) CVB3 (*n* = 30); (iii) CVB3 + MLT (*n* = 30); (iv) CVB3 + MLT + 3-methyladenine (3-MA; autophagy inhibitor) (*n* = 30); and (v) CVB3 + MLT + rapamycin (RAPA; autophagy agonist) (*n* = 30). Mice in the normal group were injected daily with phosphate-buffered saline (PBS), and all other groups were infected with 0.1 ml CVB3 (10^5.5^ TCID_50_). The day of virus inoculation was defined as day 0. The mice received intraperitoneal injections of PBS, MLT (14.4 mg/kg/day), and 3-MA (15 mg/kg/day) or RAPA (1 mg/kg/day) for 14 consecutive days, starting 24 h after viral inoculation.

### Virus Propagation and Titer Determination

Hep2 cells were maintained in Dulbecco’s modified Eagle medium supplemented with 10% fetal bovine serum at 37°C in 5% CO_2_. CVB3 (Nancy strain) was added after the cells reached 70–80% confluence. When cytopathic effects were evident, the viruses were released from the cells by at least three freeze-thaw cycles. These samples were then centrifuged at 7,500 rpm and 4°C for 15 min, and the supernatant was collected. The virus titer was calculated using the Reed–Muench formula and expressed as the half-cell infection rate (TCID_50_), measured as 10^-5.5^.

### Survival Rate

We observed survival of the mice for up to 14 days.

### Myocardial Histopathology

The heart tissue samples were fixed in 4% paraformaldehyde and embedded in paraffin. Cellular infiltration and myocardial necrosis were scored on hematoxylin and eosin-stained sections by two skilled technicians who were blinded to the experimental treatments. Severity was assessed as follows: 0 = no lesion; 1+ = lesion involving <25% of the myocardium; 2+ = 25–50%; 3+ = 50–75%; and 4+ = 75–100% (Li-Sha et al., 2017).

### Western Blot Analysis of Apoptosis and Autophagy-Related Proteins

The expression levels of Bcl-2, Bax, cleaved caspase-9, cleaved caspase-3, LC3I/II, beclin-1, p62, and β-actin were measured by western blot. The proteins in each sample were extracted from heart tissue, and the protein concentration was determined with a BCA protein assay. Then, 40 μg of protein from each sample was separated by 12% sodium dodecyl sulfate-polyacrylamide gel electrophoresis and transferred to a polyvinylidene difluoride membrane. The membranes were blocked in 5% fat-free milk and incubated with the appropriate primary antibodies overnight followed by incubation with secondary antibodies. Then, the bands were detected using an enhanced chemiluminescence (PerkinElmer, Waltham, MA, United States) kit and quantified by densitometry using AlphaEaseFC software. The primary antibodies were as follows: anti-p62, anti-Bax (1:1,000; Abcam, Cambridge, United Kingdom); anti-Bcl-2, anti-LC3I/II, anti-cleaved caspase-9, anti-cleaved caspase-3, anti-interleukin (IL-)-1, anti-tumor necrosis factor (TNF-)-α, anti-GAPDH (1:1,000; Cell Signaling Technology, Danvers, MA, United States); and anti-β-actin (1:5,000; Bioword, Shanghai, China).

### Immunohistochemical Detection of LC3-Positive Cells

Paraffin sections of heart tissue samples (4 μm thick) for LC3 analyses were dewaxed with xylene, rehydrated using a gradient ethanol series, and washed. The sections were blocked with 3% H_2_O_2_ (10 min), treated with 10.2 mM sodium citrate buffer for antigen retrieval (20 min, 95°C), and blocked in 10% (v/v) bovine serum albumin (30 min). Following overnight incubation with an antibody against LC3 (1:500; Cell Signaling Technology) at 4°C and horseradish peroxidase-conjugated secondary antibodies (2 h at 37°C), the sections were developed with DAB and hematoxylin. The integral absorbance of cells positive for LC3 was automatically counted from eight randomly selected lesion sites per sample using IPP software.

### Detection of Apoptosis

Apoptosis was detected in myocardial tissue sections using the terminal transferase-mediated DNA nick end labeling (TUNEL) assay. Apoptotic cells were identified using an *in situ* Cell Death Detection kit (POD) (Roche, Basel, Switzerland). Green staining indicated TUNEL-positive cells, whereas DAPI staining indicated the cell nucleus.

### Transmission Electron Microscopy

The myocardial tissue was placed in cold, 6% glutaraldehyde (pH 7.3) for 1 h. The samples were refrigerated for 24 h, washed in PBS (12 h), placed in cold veronal acetate (pH 7.3) containing 1% osmium tetroxide (1 h), and stained with phosphotungstic acid (10 min). Then, the samples were embedded in Ciba 502 by polymerization (35°C, 12 h; 45°C, 8 h; 60°C, 12 h). The sections (60–70 nm) were stained with toluidine blue and placed on carbon-coated 200 mesh grids. An RCA EMU-3F electron microscope at 50 KV was used to examine the tissues.

### Doppler Echocardiography

Transthoracic echocardiography was performed on day 14 with an M-mode transducer (12-MHz phased-array transducer; Sonos 5500, Philips USA, Bothell, WA, United States). The short-axis view of the M-mode tracing was recorded at the papillary muscle level through the anterior and posterior left ventricular (LV) walls to measure LV end-diastolic dimension (LVEDd) and the LV end-systolic dimension (LVESd) over the course of at least three consecutive cardiac cycles. The LV ejection fraction (EF) and fraction shortening (FS) were calculated using these measurements. An experienced technician who was blinded to the study groups performed all measurements.

### Statistical Analysis

All statistical analyses were performed using SPSS 20 software. Mean values ± SEs were used to express the data. Multiple comparisons were performed using a one-way analysis of variance (ANOVA), followed by a Fishers least significant difference test. The results were compared using Kruskal–Wallis *H* test. *P* < 0.05 was considered significant.

## Results

### Survival Rate

**FIGURE 1:**
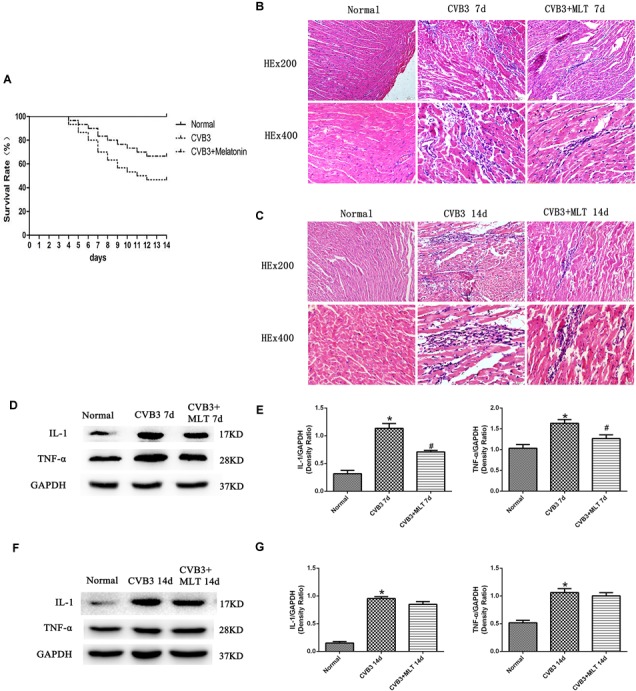
The survival rates of three groups of mice followed for 14 days, with HE staining of the myocardial tissue to evaluate the histological changes on days 7 and 14 (*n* = 5 in each group, three sections per heart were examined), and the expression of proinflammatory cytokines in the myocardium (*n* = 5 in each group). **(A)** Compared to the CVB3 group, the survival rate of the CVB3 + MLT group was increased. **(B,C)** Histological analysis of the infected hearts on days 7 and 14 (original magnification × 200, × 400). **(D,F)** Representative levels of proinflammatory cytokines on days 7 and 14. **(E,G)** Density ratio of the proinflammatory cytokines to Gapdh. ^∗^*P* < 0.05 versus the normal group, ^#^*P* < 0.05 versus the CVB3 group.

No deaths occurred in any animal in the normal group. Fourteen of 30 mice survived in the model group (survival rate: 46.7%), and 20 of 30 mice survived in the MLT treatment group (survival rate: 67.7%) (*P* < 0.05) (Figure 1A).

### MLT Alleviates Myocardial Injury in CVB3-Induced Myocarditis

The histopathological analysis of samples revealed that obvious myocardial injury in the CVB3-induced model group on days 7 and 14 compared to the normal group. These results indicated inflammatory cell infiltration, necrosis, and interstitial edema in CVB3-induced myocarditis. MLT administration alleviated the severity of the myocarditis (Tables 1, 2). There were no significant differences in the myocardial injury among the CVB3 + MLT, CVB3 + MLT + 3-MA, and CVB3 + MLT + RAPA groups on days 7 and 14. Representative myocardial pathological changes are shown in Figures 1B,C, 5A, 6A.

**Table 1:** Myocardial pathological score of the three groups.

	*n*	Infiltration		Necrosis	
		7 day	14 day	7 day	14 day
Normal	5	ND	ND	ND	ND
CVB3	5	2.2 ± 0.1	1.7 ± 0.2	1.2 ± 0.1	1.6 ± 0.1
CVB3+MLT	5	1.5 ± 0.2^∗^	1.0 ± 0.2^∗^	0.9 ± 0.1^∗^	1.3 ± 0.2^∗^

*ND, not detected. ^∗^*P* < 0.05, versus CVB3*.

**Table 2:** Myocardial pathological score of the five groups.

	*n*	Infiltration		Necrosis	
		7 day	14 day	7 day	14 day
Normal	5	ND	ND	ND	ND
CVB3	5	2.9 ± 0.1	2.5 ± 0.2	1.6 ± 0.2	1.8 ± 0.1
CVB3+MLT	5	1.4 ± 0.2^∗^	1.3 ± 0.1^∗^	0.8 ± 0.1^∗^	1.0 ± 0.2^∗^
CVB3+MLT+3-MA	5	1.6 ± 0.2^∗^	1.1 ± 0.1^∗^	1.0 ± 0.2^∗^	1.1 ± 0.2^∗^
CVB3+MLT+RAPA	5	1.2 ± 0.2^∗^	1.5 ± 0.2^∗^	0.7 ± 0.1^∗^	1.2 ± 0.1^∗^

*ND, not detected. ^∗^*P* < 0.05, versus CVB3*.

### Levels of Proinflammatory Cytokines in the Heart on Days 7 and 14

We detected the expression of proinflammatory cytokines from each group on days 7 and 14. The expression levels of IL-1 and TNF-α were higher in the CVB3 group than the normal group on days 7 and 14 (*P* < 0.05). MLT reduced the production of proinflammatory cytokines in the CVB3 + MLT group on day 7 (CVB3 + MLT versus CVB3, *P* < 0.05). No significant differences were observed in the levels of proinflammatory cytokines among the CVB3, CVB3 + MLT, CVB3 + MLT + 3-MA, and CVB3 + MLT + RAPA groups on day 14 (Figures 1D–G, 5B, 6B).

### MLT Decreases Microstructural Injury and Alters Autophagy in CVB3-Induced Myocarditis

**FIGURE 2:**
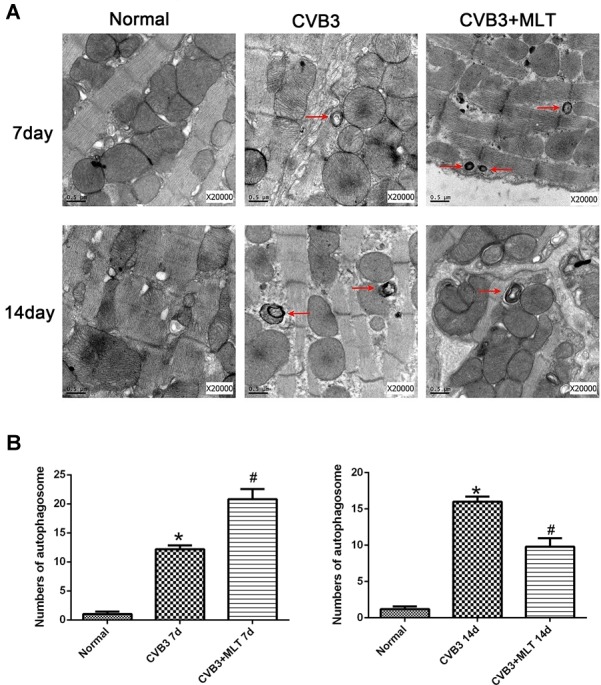
Transmission electron microscopy showing the myocardial microstructure and autophagosomes (red arrowheads) on days 7 and 14 (scale bars = 0.5 μm). **(A)** Representative transmission electron microscopy image. **(B)** The results of the statistical analysis of the numbers of autophagosomes from 20 fields (*n* = 5 hearts per group). ^∗^*P* < 0.05 versus the normal group, ^#^*P* < 0.05 versus the CVB3 group.

We used TEM to observe myocardial microstructure, autophagosomes, and autolysosomes. The myocardial cells in the normal group had integrated membranes and cytoplasm, which appeared full of aligned myofibrils divided neatly into sarcomeres by the Z line. The ultrastructure of the CVB3-induced myocardial cells was broken, accompanied by round or oval shaped mitochondria. In contrast, this condition was ameliorated in the CVB3 + MLT group (Figure 2A). Additionally, the CVB3 + MLT group had an increased number of autophagosomes on day 7 and a decreased number on day 14 compared with the CVB3 group (*P* < 0.05) (Figures 2A,B).

### Levels of Autophagy in MLT-Treated CVB3-Induced Myocarditis on Days 7 and 14

**FIGURE 3:**
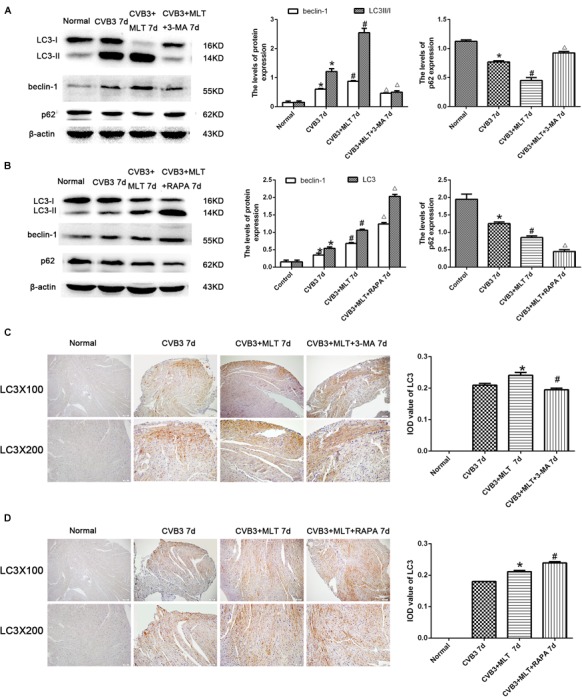
Levels of autophagy in the heart on day 7 (*n* = 5 in each group). **(A,B)** Expression of autophagy-related proteins in the myocardial tissues of mice in four groups on day 7. **(C,D)** Immunohistochemistry for LC3 on day 7. ^∗^*P* < 0.05 versus the normal group, ^#^*P* < 0.05 versus the CVB3 group, ∆*P* < 0.05 versus the CVB3 + MLT group.

**FIGURE 4:**
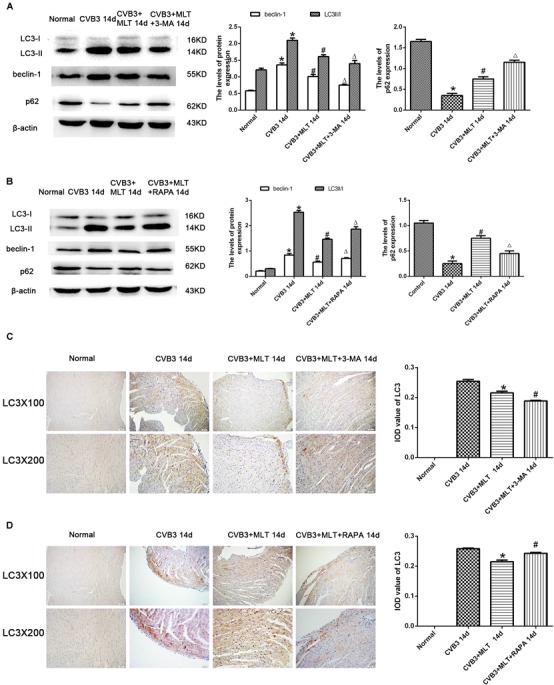
Levels of autophagy in the heart on day 14 (*n* = 5 in each group). **(A,B)** Protein expression levels of LC3II/I, Beclin1, and p62 in myocardial tissue of mice in four groups on day 14. **(C,D)** Immunohistochemistry for LC3 on day 14. ^∗^*P* < 0.05 versus the normal group, ^#^*P* < 0.05 versus the CVB3 group, ∆*P* < 0.05 versus the CVB3 + MLT group.

The autophagy inhibitor 3-MA and autophagy agonist RAPA were administered to evaluate the levels of autophagy in MLT-treated CVB3-induced myocarditis, and immunohistochemistry was used to detect the autophagosomal marker LC3II in the affected cells. LC3II expression increased in the CVB3-induced myocarditis group compared with the normal group on days 7 and 14. The number of LC3II-positive cells increased on day 7, while the number of LC3II-positive cells decreased on day 14 in the CVB3 + MLT group compared with the CVB3 group. Furthermore, administering 3-MA significantly reduced the expression of LC3II, whereas administering RAPA significantly increased the expression of LC3II on days 7 and 14 (Figures 3C,D, 4C,D). Expression of the LC3II/LC3I and beclin-1 proteins was confirmed, supporting the immunohistochemistry results (Figures 3A,B, 4A,B). Western blot analysis was used to detect p62, an autophagic substrate. The p62 level was decreased in the CVB3 group relative to the normal group on days 7 and 14. The p62 protein level decreased on day 7 and increased on day 14 in injured cardiomyocytes of the CVB3 + MLT group compared with the CVB3 group. In addition, the autophagy inhibitor 3-MA significantly increased p62 expression, while the autophagy agonist RAPA significantly decreased p62 expression on days 7 and 14 (Figures 3A,B, 4A,B). These results indicate that autophagy was significantly activated in CVB3-induced myocarditis, and that MLT treatment regulated the level of autophagy.

### Levels of Apoptosis in MLT-Treated CVB3-Induced Myocarditis on Days 7 and 14

**FIGURE 5:**
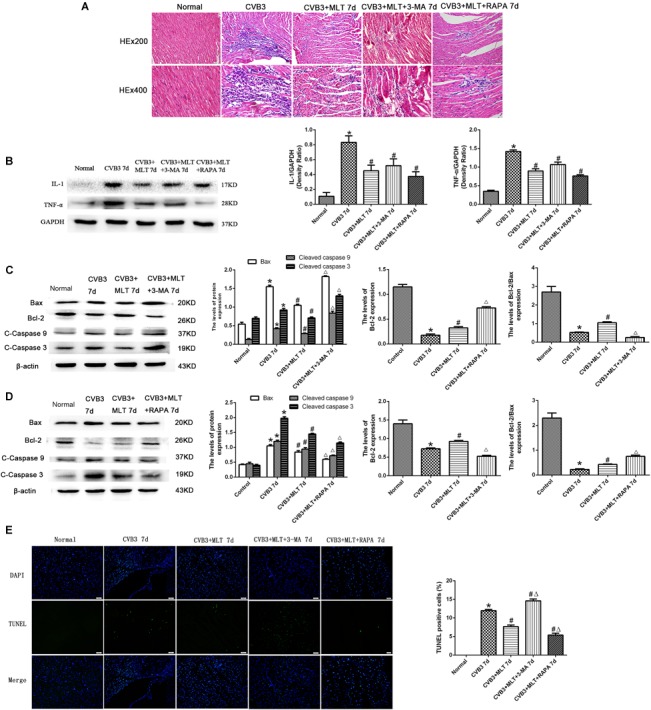
The effects of melatonin on inflammation and apoptosis on day 7. **(A)** Representative myocardial pathological changes on day 7 (*n* = 5 in each group, three sections per heart were examined). **(B–D)** Expression of proinflammatory cytokines, apoptosis-related protein Bax, Bcl-2, cleaved caspase-3, and cleaved caspase 9 in the myocardium on day 7 (*n* = 5 in each group). **(E)** Double staining for DAPI (blue) and TUNEL (green) of sections from myocardium on day 7, scale bar = 50 μm (*n* = 5 in each group). ^∗^*P* < 0.05 versus the normal group, ^#^*P* < 0.05 versus the CVB3 group, ∆*P* < 0.05 versus the CVB3 + MLT group.

**FIGURE 6:**
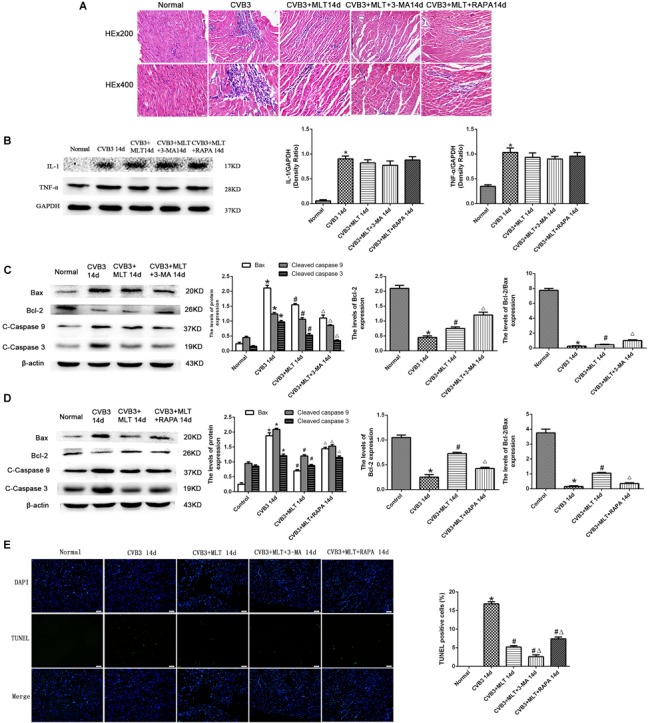
The effects of melatonin on inflammation and apoptosis on day 14. **(A)** Representative myocardial pathological changes on day 14 (*n* = 5 in each group, three sections per heart were examined). **(B–D)** Expression of proinflammatory cytokines, apoptosis-related protein Bax, Bcl-2, cleaved caspase-3, and cleaved caspase 9 in the heart on day 14 (*n* = 5 in each group). **(E)** Double staining for DAPI (blue) and TUNEL (green) of sections from myocardium on day 14, scale bar = 50 μm (*n* = 5 in each group). ^∗^*P* < 0.05 versus the normal group, ^#^*P* < 0.05 versus the CVB3 group, ∆*P* < 0.05 versus the CVB3 + MLT group.

Cleaved caspase-9, cleaved caspase-3, and Bax levels increased significantly in the CVB3 group on days 7 and 14 but were downregulated in the MLT-treated group. Cleaved caspase-9, cleaved caspase-3, and Bax levels increased in the CVB3 + MLT + 3-MA group and decreased in the CVB3 + MLT + RAPA group relative to the CVB3 + MLT group on day 7 (Figures 5C,D). Cleaved caspase-9, cleaved caspase-3, and Bax levels decreased in the CVB3 + MLT + 3-MA group compared to the CVB3 + MLT group, while they increased in the CVB3 + MLT + RAPA group on day 14 (Figures 6C,D). Bcl-2 and the Bcl-2/Bax ratio were downregulated in the CVB3 group on days 7 and 14 but increased in the MLT treatment group. Bcl-2 and the Bcl-2/Bax ratio were significantly downregulated in the CVB3 + MLT + 3-MA group but upregulated in the CVB3 + MLT + RAPA group compared with the CVB3 + MLT group on day 7 (Figures 5C,D). These results were opposite on day 14 (Figures 6C,D). Apoptosis was observed in the damaged myocardium by double staining for DAPI (blue) and TUNEL (green). MLT treatment significantly attenuated apoptosis in the CVB3 + MLT group compared with the CVB3 group on days 7 and 14 (Figures 5E, 6E). Treatment with 3-MA augmented, while RAPA treatment attenuated, the increase in apoptosis in infected mice on day 7 compared with the MLT group. These results were opposite on day 14. The results are consistent with the western blot analysis and showed that MLT attenuated the cardiac damage by activating autophagy on day 7 and inhibiting autophagy on day 14.

### MLT Improves Cardiac Function

**FIGURE 7:**
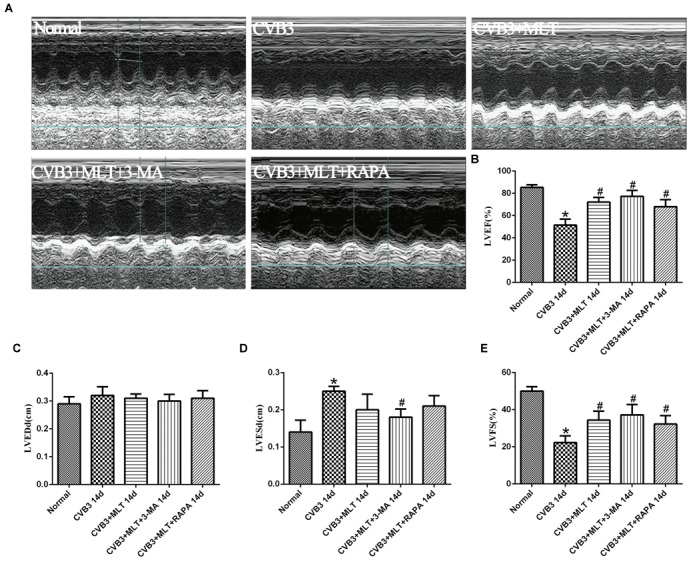
The echocardiography results in each group on day 14 (*n* = 5 in each group). **(A)** Representative M-type cardiac ultrasound image. **(B–E)** The changes of LVEF, LVEDd, LVESd, and LVFS in five groups. LVEF, left ventricular ejection fraction; LVEDd, left ventricular end-diastolic dimension; LVESd, left ventricular end-systolic dimension; LVFS, left ventricular shortening fraction. ^∗^*P* < 0.05 versus the normal group, ^#^*P* < 0.05 versus the CVB3 group.

All mice underwent echocardiography after 2 weeks. As shown in Figure 7, LVEDd was similar among the five groups. LVEF and LVFS decreased significantly 2 weeks after virus infection in the CVB3 group compared with the normal group (*P* < 0.05). The M-mode image showed weakened wall movement in the CVB3 group. MLT administration slightly decreased LVEDd and LVESd and improved LVFS and LVEF in the CVB3 + MLT group compared with the CVB3 group (*P* < 0.05). Treating the CVB3 + MLT group with 3-MA or RAPA increased LVEF and LVFS compared with the CVB3 group. No significant differences were found in LVEF, LVEDd, LVESd, or FS among the CVB3 + MLT, CVB3 + MLT + 3-MA, or CVB3 + MLT + RAPA groups (*P* > 0.05) (Figure 7).

## Discussion

In this study, we observed a marked amelioration of acute viral myocarditis following MLT treatment in a CVB3-induced murine myocarditis model. To the best of our knowledge, this is the first study to investigate the effects of MLT on viral myocarditis. We demonstrated that MLT significantly ameliorated CVB3-induced myocarditis. The therapeutic effect may be related to the regulatory effects of MLT on apoptosis and autophagy and indicate that MLT plays a beneficial role in viral myocarditis.

Melatonin is produced by the pineal gland in response to a signal generated by the master circadian clock in the suprachiasmatic nucleus of the hypothalamus, which synchronizes the phases of the circadian clock with the light–dark cycle (Venegas et al., 2012; Maria and Witt-Enderby, 2014). MLT can also act as a protective agent against pathological conditions by influencing a variety of molecular pathways, such as inflammation, apoptosis, proliferation, oxidative stress, metastasis, and angiogenesis (Maria and Witt-Enderby, 2014; Peschke et al., 2015; Reiter et al., 2016). Moreover, some studies have demonstrated a protective effect of MLT on I/R injury and myocardial infarction because of its antioxidative and anti-inflammatory activities (Hughes et al., 2015; Chua et al., 2016; Favero et al., 2016). Alberto et al. and Dwaich et al. reported that the dose of MLT administered affects reverse cardiac remodeling in patients with heart failure and those undergoing bypass surgery (Dominguez-Rodriguez et al., 2016; Dwaich et al., 2016). Several studies have proposed that MLT is a dual regulator of apoptosis and autophagy due to its important anti-inflammatory, antioxidant, and anti-apoptotic activities and for its role in the regulation of autophagy (Pollack and Leeuwenburgh, 2001).

Our results demonstrate that MLT significantly ameliorated CVB3-induced myocarditis in a mouse model with respect to improved inflammatory cell infiltration, necrosis, edema, and a decreased level of inflammation. Moreover, our data show that expression of the autophagy-related proteins LC3I/II and beclin-1 increased, and p62 decreased following MLT-treatment in CVB3-induced myocarditis on day 7, which trended the opposite way on day 14. Autophagy is a process of self-digestion (Ordonez et al., 2015). Cells degrade long-lived organelles and proteins to generate the building blocks necessary to sustain cellular function. Autophagy has protective effects on infections, neurodegenerative disorders, cancer, and inflammatory diseases and is upregulated in response to therapy, showing a survival-promoting mechanism. In contrast, autophagy can accelerate cell death in different cellular environments (Pfeifer et al., 1987; Yan et al., 2005; Miyata et al., 2006; Ordonez et al., 2015). Interestingly, CVB3 gains a replication advantage on the surface of autophagosomes by hijacking the autophagic pathway (Wong et al., 2008).

In addition, expression of the apoptosis-related proteins Bax, cleaved-caspase 3, and cleaved-caspase 9 decreased, and the anti-apoptosis protein Bcl-2 increased following MLT treatment on days 7 and 14. Apoptosis is a unique type of programmed cell death defined by cellular phenotypic changes, including nuclear chromatin condensation, DNA fragmentation, caspase activation, cell shrinkage and membrane blebbing (Kerr et al., 1972). Previous studies have shown that apoptosis occurs *in vitro* and *in vivo* in response to CVB3 infection and is a hallmark of CVB3-induced pathogenesis (Carthy et al., 1998). However, the role of apoptosis in CVB3-induced myocarditis remains unclear (Cunningham et al., 2003).

Autophagy and apoptosis are directly related (Rubinsztein et al., 2015) and the balance between autophagy, apoptosis, and necrosis is crucial for therapeutic interventions (Xu et al., 2005). Previous studies have shown that autophagy is protective during ischemia, but detrimental during reperfusion, which plays a dual function in I/R (Matsui et al., 2007). Excessive autophagic activity can destroy a large fraction of the cytoplasm and organelles, especially mitochondria and the endoplasmic reticulum, resulting in abnormal cell morphology, apoptosis, necrosis, and cellular dysfunction (Nishida et al., 2008).

Finally, MLT administration improved cardiac function and survival. These results demonstrate that MLT plays a dual role preventing apoptosis by adjusting the levels of autophagy at different stages of viral myocarditis. Some studies have reported that autophagy has a twofold effect on CVB3. Autophagy can clear a small number of CVB3 viruses (Wong et al., 2008), whereas autophagy sustains the life cycle of the CVB3 virus (Robinson et al., 2014). Interestingly, it is not known how CVB3 regulates the levels of autophagosomes. First, Wong et al. (2008) reported that CVB3 could be removed via autophagy by inhibiting LAMP-2, a lysosomal membrane protein critical for autophagosome-lysosome fusion, which enhances CVB3 replication. Second, the expression of core autophagy components suppresses CVB3 replication (Wong et al., 2008; An et al., 2012). Third, Robinson et al. (2014) reported that autophagy plays a vital role in the shedding and release of CVB3. Thus, we postulate that autophagy is closely related to CVB3 replication and release; however, the mechanism should be identified in future studies.

Some limitations of this study should be discussed. The model was run in the setting of disease development (prevention mode) and not disease treatment. The dose-response and the side effects of MLT should be determined before it is used in a clinical setting. Although we found that autophagy and apoptosis may be involved in MLT-mediated cardiac protection, other mechanisms should also be identified in future studies.

## Conclusion

Viral myocarditis remains a challenging disease, and novel effective therapies based on fundamental mechanisms are needed in clinical practice. In this study, MLT treatment dramatically alleviated CVB3-induced myocarditis, improved LV function, and regulated the levels of autophagy and apoptosis. Thus, MLT may be a promising medication for viral myocarditis therapy, and further experimental and clinical studies are necessary.

## Author Contributions

MC and YZ designed the whole study. YS, XG, LP, CZ, XR, TW, TX, YL, and LG performed the experiments. YS, XG, and LP wrote the paper.

## Conflict of Interest Statement

The authors declare that the research was conducted in the absence of any commercial or financial relationships that could be construed as a potential conflict of interest.
